# Considerations for Better Management of Postoperative Pain in Light of Chronic Postoperative Pain: A Narrative Review

**DOI:** 10.7759/cureus.23763

**Published:** 2022-04-02

**Authors:** Maria Gómez, Cesar E Izquierdo, Victor Mayoral Rojals, Joseph Pergolizzi Jr, Ricardo Plancarte Sanchez, Antonella Paladini, Giustino Varrassi

**Affiliations:** 1 Federación Latinoamericana de Asociaciones para el Estudio del Dolor, Hospital Universitario Nacional de Colombia, Bogotá, COL; 2 Pain Management and Palliative Care, Instituto Nacional de Salud del Nino, San Borja, PER; 3 Anesthesiology, Bellvitge Hospital, L'Hospitalet de Llobregat, ESP; 4 Pain Medicine and Critical Care Medicine, Nema Research, Inc., Naples, USA; 5 Anesthesiology, University Autonomous of Mexico, Mexico City, MEX; 6 Department of Life, Health and Environmental Sciences (MESVA), University of L'Aquila, L'Aquila, ITA; 7 Pain Management, Paolo Procacci Foundation, Rome, ITA

**Keywords:** prevention of chronic postoperative pain, enhanced recovery after surgery, acute postoperative pain, perioperative analgesia, chronic postoperative pain

## Abstract

Chronic postoperative pain (CPOP) is prevalent, with particularly high rates in breast surgery, thoracotomy, and amputation. As the world emerges from the coronavirus disease 2019 (COVID-19) lockdowns, it is expected that there will be an increase in surgical procedures, elevating the importance of preventing CPOP in the coming years. Risk factors are emerging to better stratify patients at high risk for CPOP. Perioperative analgesia plays an important role in managing acute postoperative pain and in some cases may limit its transition to CPOP. Acute postoperative pain is adaptive, normal, expected, and has a well-defined trajectory, while CPOP is maladaptive and, as a form of chronic pain, is challenging to treat. Good analgesia, early ambulation, and rehabilitation efforts may be helpful in preventing CPOP following certain surgeries. Enhanced Recovery After Surgery (ERAS) protocols present guidance to help promote recovery and prevent CPOP.

## Introduction and background

Acute postoperative pain is the expected consequence of certain surgical procedures and because pain diminishes as the tissue heals, it has a predictable trajectory. Chronic postoperative pain (CPOP) has been defined as pain persisting after surgery and for which other causes, such as pre-existing pain, can be excluded [1]. In a subpopulation of surgical patients, CPOP develops, affecting about 20-30% of surgical patients, and it is the reason for about 20% of consultations at pain clinics [2]. The impact of CPOP on the patient may range from mild to severe pain and from discomfort to dysfunction [3]. Despite its prevalence, CPOP has not been widely studied, with the result that the incidence of CPOP has not changed much in the past decades [4]. While the transition from acute to chronic postsurgical pain is often described in temporal terms, profound and fundamental physiologic changes are at play [5].

While certain surgery-related and patient-specific risk factors for CPOP have emerged, it appears crucial that acute postoperative pain control is essential to mitigate the risk for CPOP [4]. To better control acute postoperative pain, a patient-centric approach is needed. Furthermore, knowledge gaps in our understanding of the transition of acute pain into chronic pain need to be filled along with the development of effective mitigation strategies.

## Review

Epidemiology and risk factors

It is difficult to state one figure for the incidence of CPOP, as the rate of this condition varies among patients and procedures. For example, the incidence of CPOP is particularly high following limb amputation and thoracotomy [6]. Moreover, the elucidation of CPOP has been hampered by the fact that there are few studies of the condition that exists in multiple forms with many variables, and comparing studies has been impeded by the fact that they sometimes use different definitions of CPOP [7].

Although multiple definitions appear in the literature [4], CPOP will be defined here as pain that was not pre-existing before surgery and that persists for more than or equal to three months after surgery [1]. CPOP may be localized to the surgical site and its immediate vicinity, or it may be referred to distant parts of the body. Any diagnosis of CPOP must exclude all other potential sources of such pain [8]. CPOP may occur following an otherwise successful surgery and is not indicative of surgical failure [3]. CPOP represents a substantial burden to patients and may cause functional deficits, economic hardships, reduced quality of life, family stress, and suffering [7].

An increase in surgical procedures is anticipated as the coronavirus disease 2019 (COVID-19) pandemic ends [9]. The CPOP population is already large; with 300 million surgeries per year around the world and a 30% average incidence of CPOP, the current incidence of CPOP may be as high as 90 million people [10]. An increase in surgical procedures will likely mean clinicians will be treating many new CPOP patients.

CPOP often has a neuropathic component, making it particularly challenging to treat [11]. In a study of surgical patients evaluated 12 months after surgery, 11.8% of patients reported persistent moderate to severe pain (defined as pain rated ≥6 on a 0-10 scale), and of those patients with CPOP, 35.4-57.1% had signs of neuropathic pain [12]. A neuropathic pain component with CPOP has been associated with mastectomy, thoracotomy, hernia, and arthroplasty procedures [12]. Besides the type of surgery, pre-existing pain, particularly of long duration, preoperative opioid use [12], and mental health conditions, specifically anxiety, depression, catastrophizing, and fear of postoperative pain, may also confer risk for CPOP [13]. The duration of surgery appears to play a role, but CPOP may occur after minor as well as major operations [12]. Females have a greater risk of developing CPOP than males, and younger patients have a greater risk than older patients [14]. In a study of 2,929 surgical patients, CPOP developed in 18.0% of patients overall, but no significant genetic markers for predicting CPOP risk emerged [15]. In a study of 911 surgical patients at a level one trauma center, 14.8% of patients reported CPOP at one year, with the most frequently named pain sites being the joints (49.4%), the incision or scar (37.7%), and nerve pain (33.7%) [16]. In the first 24 hours after surgery, the duration of severe pain may be associated with the risk of developing CPOP; for every 10% increase in time spent in severe pain in that “first 24” window of time, the risk of developing CPOP increases by 30% [14]. It is interesting to note that pain intensity overall was not predictive for CPOP, only the duration of severe pain in the “first 24.”

Preoperative pain sensitivity may also predict CPOP, and preoperative quantitative sensory testing may turn out to be a useful predictor of CPOP risk [17]. Patients with heat hyperalgesia prior to surgery consumed more morphine after surgery than those without heat hyperalgesia [18]. Cross-sectional studies suggest that preoperative pain may increase the risk of CPOP by two or threefold [19,20].

Murine studies identified certain receptors, mediators, and neurotransmitters involved in peripheral and central sensitization following surgical incision, confirming that some were unique and specific to incisional pain [21]. The size, type, and location of the incision resulted in different pain states and different analgesic effectiveness. Therefore, pain management must account for the specific type and location of surgery. These preclinical studies also suggest that neuropathic pain can be acute as well as chronic [21]. Epigenetic modulation, which includes DNA methylation, histone acetylation, and non-coding RNA, may hold promise for managing chronic pain and there are preclinical studies evaluating epigenetic modulation for acute postoperative incisional pain. This is based on the finding that an incision appears to provoke changes in DNA methylation [22]. This, in turn, leads to hyperalgesia at the incision site. In animal studies, peripheral and spinal inhibition of DNA methyltransferase by way of 5-Aza-2’-deoxycytidine reduced mechanical pain, heat hyperalgesia, and swelling [22]. Since epigenetic findings suggest that both peripheral and central epigenetic modulation play a role in nociceptive sensitization after surgical incision, it seems reasonable to postulate that drugs that affect epigenetic regulation, such as opioids, along with environmental factors, may trigger what could be long-term changes to the body’s pain system, even sufficient to transition an acute pain state into chronic pain [21].

Analgesics for acute postoperative pain

The International Association for the Study of Pain (IASP) declared 2017 to be the global year against pain after surgery to raise awareness about this pain state and encourage policies to advance better treatment and clinician education [23]. In particular, the IASP recommended a multimodal analgesic approach, when feasible, for acute postoperative pain, and individualized care such that pain control is integrated into other aspects of pre- and post-surgical care [23]. Multimodal analgesia utilizes two or more agents with complementary mechanisms of action to target two or more pain mechanisms. Acute postoperative pain may be treated with monotherapy, such as opioid analgesia, but multimodal regimens can be equally effective, have fewer or milder side effects, and spare opioid consumption [24]. In fact, one of the goals of multimodal analgesia is to reduce total opioid consumption without sacrificing analgesic benefits. This can be accomplished using local anesthetics (when appropriate), nonopioid pain relievers such as non-steroidal anti-inflammatory drugs (NSAIDs), gabapentinoids, N-methyl-D-aspartate (NMDA) receptor antagonists such as ketamine, alpha-2 adrenergic agents such as clonidine, and anti-inflammatory agents such as corticosteroids [25,26].

The role of ketamine in managing surgical pain is expanding, and it is a field of current investigation [27]. In a study of 40 patients who underwent intravenous (IV) regional anesthesia for intraoperative or postoperative pain control, patients were randomized to receive lidocaine 3 mg/kg alone or lidocaine plus ketamine 50 mg. The ketamine patients required significantly less postoperative analgesia and had greater patient satisfaction than the control group [28]. Perioperative low-dose administration of ketamine may reduce postsurgical pain and postsurgical opioid consumption [29]. A meta-analysis of bariatric surgery patients found that perioperative IV ketamine improved pain outcomes following surgery, although ketamine did not confer late-postoperative analgesic benefits [30].

Gabapentinoids, such as pregabalin and gabapentin, are gamma-aminobutyric acid (GABA) analogs active at the NMDA receptors. Gabapentinoids bind at the voltage-gated Ca2+ channel subunits, which are proteins that interact with the NMDA receptors and increase their synaptic activity, leading to neuropathic pain. While the exact mechanism of action is not yet elucidated, it appears that gabapentinoids can inhibit the anterograde trafficking of these subunits from the dorsal root ganglion to the dorsal horn neurons and, in this way, reduce neuropathic pain [31]. Gabapentinoids can be effective in treating both chronic neuropathic pain and acute postoperative pain [32]. The use of gabapentinoids following surgery may decrease the rate of chronic postoperative pain [33]. Increased endogenous inhibitory modulation may be an important aspect of the use of gabapentinoids in postoperative pain control [34].

There has yet to be a consensus about the use of perioperative ketamine and postoperative gabapentinoids for the control of surgical pain [26]. There is an incomplete understanding of the mechanism of action of ketamine and a very narrow therapeutic window [29]. Gabapentinoids may cause dizziness, somnolence, drowsiness, blurred vision, ataxia, long-term weight gain, and other conditions [35]. Both ketamine and gabapentinoids can be misused [36].

Chronic inflammation can initiate neuroplastic changes that result in aberrant pain signaling known as sensitization, which may result in CPOP [37].

Regional anesthesia

Regional anesthesia may offer benefits beyond better acute pain control. In a retrospective study of patients undergoing primary hip or knee arthroplasty (n = 382,236), 74.8% of patients had only general anesthesia, while 11% had neuraxial anesthesia, and 14.2% had a combination. Patients who had neuraxial anesthesia or the combination of neuraxial plus general anesthesia had significantly better outcomes in 30-day mortality along with shorter hospital lengths of stay, lower in-hospital complication rates, and reduced costs [38]. Similar findings emerged in a retrospective study of orthopedic surgery patients in which spinal anesthesia was associated with a shorter length of stay than general anesthesia (5.7 vs. 6.6 days, p < 0.001) and a significantly lower 30-day mortality rate [39]. A meta-analysis found moderate-quality evidence that regional analgesia reduced the risk of CPOP 3-18 months after surgery following thoracotomy and 3-12 months following cesarean section [40]. The evidence supporting regional analgesia for preventing postoperative pain 3-12 months after breast cancer surgery was low quality, but moderate-quality evidence showed an IV infusion of local anesthetics reduced the risk of CPOP for three to six months after breast cancer surgery [40]. Regional anesthesia may offer other advantages in oncologic procedures [41], but the mechanistic evidence that regional anesthesia may inhibit tumor cell seeding by inhibiting the adrenergic and inflammatory responses to surgery is inconclusive [42].

There are essentially three potential injection sites for regional anesthesia: the perineural and sub-perineural region, the sub-epineural area, and the intrafascicular. The perineural region is outside the nerve itself, the sub-epineural region is below the epineurium, and the intrafascicular injection injects the anesthetic directly into the fascicles containing nerve fibers within the perineurium [43]. The use of ultrasound imaging has vastly expanded the safety and utility of regional anesthesia [44]. It was only with the widespread availability of high-quality ultrasonography technology that complex injections such as supraclavicular brachial plexus block became more widely used [45]. The use of real-time ultrasound guidance during needle advancement has been described in the literature [45]. Optimal needling techniques are still being studied and 14% of patients undergoing supraclavicular blocks may still experience neurological symptoms up to one week after operation [46]. In a cadaver study, an echogenic needle using the in-plane technique was used to target a cluster of hypoechoic structures corresponding to neural tissue to evaluate the rate of sub-perineural needle placement in a single injection into the supraclavicular fossa [47]. The ink was used to allow for subsequent histological evaluation. The incidence of ink deposits was extra-epineural in 32% of cases, sub-epineural but outside the perineurium in 44%, and sub-perineural in 24% of cases, raising safety concerns [47]. In a clinical study, 257 patients undergoing outpatient shoulder arthroscopy were sedated and administered ultrasound-guided interscalene blockade (n = 122) or supraclavicular block (n = 135) [48]. Patients were followed at one week and again after four to six weeks following surgery. Intraneural injection occurred in 17% of patients (n = 24) and no patient experienced neurological complications at follow-up [48].

It is not clear if neurological insult sustained during regional anesthesia is caused by nerve puncture and/or intraneural injection [49]. The optimal needle approach for avoiding epineural penetration remains to be clarified and operator experience plays a role in procedural success [49]. A tangential approach is associated with less risk of intraneural penetration [49].

Hemidiaphragmatic paresis may occur in up to 100% of patients undergoing ultrasound-guided interscalene brachial plexus block [50]. In a study of 40 patients randomized to undergo conventional and extrafascial injection, 21% of the extrafascial group and 90% of the conventional group experienced hemidiaphragmatic paresis (p < 0.0001). Extrafascial patients also had better respiratory outcomes, longer time to first request for opioid analgesics, and lower pain intensity scores 24 hours after surgery [50].

Fascial plane blocks allow needle insertion and injection into two separate fascial layers, which permits the local anesthetic to spread to the nerves in that plane [51]. Known to be effective in thoracic and abdominal surgeries, fascial plane blocks are associated with few adverse events [44]. They may be used in patients with coagulation disorders and can serve as an opioid-sparing technique. A transversus abdominis plane (TAP) block is used between the internal oblique and the transversus abdominis muscles as a field block. Ultrasound-guided quadratus lumborum blocks, erector spinae plane blocks, pectoralis (PECS) and serratus nerve blocks, serratus plane blocks, mid-point transverse process to pleura block, and retrolaminar blocks may supplement the TAP block [44]. Ultrasound-guided interfascial plane blocks are a promising new development in local anesthesia, but more investigation is needed to better identify the appropriate patients for this technique. In particular, a better understanding is needed of fascia, a lattice-like network of collagen fibers filled with a hydrated glycosaminoglycan matrix containing adipocytes and fibroblasts; fascia allows fluids to pass through but blocks heavy flows [51]. Fascial plane blocks are analgesic rather than anesthetic techniques. The mechanism of action remains to be elucidated but two prominent hypotheses have emerged. The first is that the injected drug disperses itself in the fascial plane, adjacent muscles, and into tissue compartments, and, in that way, affects the nociceptors and neurons. The other explanation is that the agent is absorbed by the vasculature and provides a systemic analgesic effect at distal sites. Regardless of the mechanism of action, fascial plane blocks can be unpredictable in terms of the extent of spread and the cutaneous sensory loss [52]. Ultrasound-guided fascial plane blocks can be important alternatives to thoracic epidural or paravertebral blockade particularly in breast surgery. Navigational landmarks are particularly important in this technique, which is generally safe and well-tolerated [53].

In a systematic review and meta-analysis comparing pectoral nerve type 2 (PECS2) analgesic block to systemic analgesia alone and with a thoracic paravertebral block in breast cancer surgery (13 trials, n = 815), the primary endpoint was postoperative opioid consumption in the first 24 hours after surgery and secondary outcomes included pain scores at zero, three, six, nine, and 24 hours following surgery, time to first request for a pain reliever, and postoperative nausea and vomiting. Compared to systemic analgesia alone, the PECS2 block significantly reduced postoperative opioid consumption and decreased acute pain intensity at all time points in the first 24 hours. PECS2 and thoracic paravertebral block had similar opioid consumption after surgery and similar pain scores. Thus, PECS2 offered more effective analgesia to breast cancer patients than systemic analgesia alone and comparably effective analgesia with respect to a thoracic paravertebral block [54].

In a retrospective cohort study of 79 patients who underwent erector spinae plane block for treatment of multiple rib fractures, pain scores were reduced from a mean of 7.7 to 4.7 in the first three hours (p < 0.01), no changes were observed in the mean arterial blood pressure versus baseline, and incentive spirometry volumes improved from 784 to 1,375 ml (p < 0.01) over the first 24 hours with the block [55]. Thus, patients treated with erector spinae plane block had improved inspiratory capacity, and better analgesia, without any hemodynamic instability. The main types of anterolateral and posterior chest wall blocks are summarized in Table 1 [53].

**Table 1:** Types of chest wall blocks and their neural targets.

Name	Technique	Neural targets
Pectoral nerve type 1 block (PECS1)	Injection between the pectoralis major and minor muscles	Lateral and medial pectoral nerves
Pectoral nerve type 2 block (PECS2)	PECS1 plus a second injection deep into the pectoralis major and superficial to serratus anterior muscle	Lateral and medial pectoral nerves. Lateral cutaneous branches of the T2-T6 intercostal nerves
Serratus anterior plane block (superficial)	Injection superficial to the serratus anterior muscle anywhere with the area bounded by the anterior and posterior axillary lines and the 3rd to 6th ribs	Lateral cutaneous branches of the T2-T7 intercostal nerves
Serratus anterior plane block (deep)	Injection deep to the serratus anterior muscle and superficial to ribs and intercostal muscles anywhere in the area bounded by the anterior and posterior axillary lines and the 3rd to 6th ribs	Lateral cutaneous branches of the T2-T7 intercostal nerves. This approach may reach the intercostal nerves as well
Retrolaminar block	Injection in the plane between the bony vertebral lamina and overlying muscles (transversospinalis and erector spinae muscle groups)	Dorsal and ventral rami of the spinal nerves, extending ~1 to 3 levels cranial and caudal with respect to the level of injection
Erector spinae block	Injection in the plane between the bony vertebral transverse processes and the overlying erector spinae muscle (longissimus thoracis)	Dorsal and ventral rami of spinal nerves, extending ~1 to 3 levels cranial and caudal with respect to the level of injection
Midpoint transverse process-to-pleura block (MTP)	Anterior injection deep into the posterior aspect of the vertebral transverse process but superficial to the superior costotransverse ligament, in other words, the needle tip does not enter into the paravertebral space	Dorsal and ventral rami of spinal nerves, extending ~1 to 3 levels cranial and caudal with respect to the level of injection
Paraspinal-intercostal plane blocks	Injection in the plane between the bony ribs plus the intercostal muscles and the overlying erector spinae muscle (the longissimus thoracis or the iliocostalis muscle, depending on how far lateral to the midline the injection site is)	Lateral cutaneous branches of T2-T7 intercostal nerves
Rhomboid intercostal subserratus plane block (RISS)	Initial injection to the plane between the bony ribs then to intercostal muscles and overlying rhomboid major muscle plus erector spinae muscle	Lateral cutaneous branches of T2-T7 intercostal nerves with potential extension as far as the T10 dermatome

An important consideration with any regional anesthesia is the duration of effect. Continuous catheterization can help sustain durable results but is limited to the ability to maintain the catheter in place. While the catheter is in place, continuous catheterization allows for flexibility in analgesia, prolonging the duration and increasing the intensity of pain relief as needed; it may also mitigate rebound pain. The main drawback to continuous catheterization is that insertions take time and precision, it may be complex to maintain throughout surgery, and it likely requires referral to acute pain care for follow-up [44]. Other risks include infection, prolonged blockage, and potential catheter migration. Liposomal bupivacaine is a sustained-release form of local anesthetic and the estimated duration of effect from a single injection is 24-72 hours. It is quick and straightforward to administer [44]. However, liposomal bupivacaine is only approved for interscalene brachial plexus block, TAP block, and surgical infiltration. Only limited evidence supports its use, and costs can be prohibitive because liposomal bupivacaine costs approximately 200 times more than ordinary bupivacaine [44]. Off-label local anesthetic adjuvant agents are sometimes used, and they may extend the effects of blockade for six to eight hours [44]. From a clinical study of 80 patients evaluating catheters placed in the popliteal-sciatic perineural region, ultrasound guidance was shown to be effective, saved time, and resulted in fewer placement failures compared to the use of older stimulating catheters, although the latter provides modestly improved analgesia [56].

Modifiable risk factors

A prospective multicenter cohort study evaluated 2,929 patients undergoing inguinal hernia repair, hysterectomy (vaginal or abdominal), or thoracotomy, with the primary endpoint being the presence of CPOP at four months following surgery; CPOP was also measured at 12 and 24 months [15]. In a median of 4.4 months, CPOP was reported by 18.0% of patients. The rates of CPOP varied by surgery with 13.6% after hernia repair, 11.8% after vaginal hysterectomy, 25.1% following abdominal hysterectomy, and 37.6% after thoracotomy. Six clinical predictors for CPOP emerged: the type of surgery, patient’s age, patient’s physical and mental health, the pre-existence of pain within the surgical field, and preoperative pain at another site(s) [15]. Four of these predictors are potentially modifiable factors: the physical and mental health of the patient and preoperative pain either within the surgical field or at other locations. While the incidence of CPOP may occur in 50% or more patients after certain surgeries, severe CPOP at one year occurs in approximately 5-10% [19,37,57,58]. Determining factors for severe CPOP include younger age, female sex, catastrophizing, hypervigilant personality, high response to evoked pain, diffuse preoperative pain, neuropathic pain, and extensive tissue damage, although the latter was not always predictive of severe-intensity pain [59,60].

Early mobilization

Early mobilization is important to recovery and is only possible when good analgesia has been delivered. Knowing the value of early mobilizations, clinicians are better equipped to help improve perioperative patient status by enhancing the patient’s physical function, addressing their mental status, and relieving pre-existing pain. Note that long ago, patients and their families received little to no information about the perioperative process. Large scars, prolonged hospital stays, and pain medications given on an as-needed basis were not just the norm, but they were expected and accepted. Apart from simple breathing exercises, patients were more or less expected to stay in bed and passively await recovery. In the past decades, the paradigm has radically shifted. Today, patients and their families are expected to be active participants in care, to be informed about the operation, to share decision-making, and to understand the recovery process and its milestones. With these changes, faster recovery times, earlier discharges, and more ambulatory procedures have become the norm. Minimally invasive surgeries, regional analgesia, and multimodal pain control are increasingly common. Despite this major shift in caring for surgical patients, a study of 45 patients in a veterans’ hospital (100% male, mean age of 74.2 years, all able to walk) with a mean hospital stay of 5.1 days found that patients spent, on average, 83% of their time in bed. The time they spent standing or walking ranged from 0.2% to 21% of the time for a mean of 43 minutes per day [61].

Exercise before surgery has been found to be effective in reducing hospital length of stay and complication rates following cardiac surgery and this benefit appears most pronounced in vulnerable patients [62]. Appropriate postoperative exercise and rehabilitation should start as soon as possible following surgery, with the primary goal of regaining functional mobility [62]. Henrik Kehlet, Chief of Surgery and Professor of Surgery at Copenhagen University, has been a pioneer in this paradigm shift about surgical recovery, stressing early mobilization after surgery [63]. The benefits of early ambulation are many: reduction in sarcopenia, atelectasis, and pneumonia, venous thromboembolism and improvements in cardiovascular fitness, insulin sensitivity, glucose effectiveness, muscle capillary density, and muscle lipid oxidation [62].

The Enhanced Recovery After Surgery (ERAS) protocol defines a multimodal course of evidence-based practices for perioperative patient care with the goal of improved outcomes. ERAS is a multidisciplinary approach and ERAS guidance has been published for specific surgeries [64]. While early mobilization has been a pillar of the ERAS protocols, it is only part of a larger picture. Preoperatively, patients should practice fluid and carbohydrate loading, have antibiotic prophylaxis, and be educated about what to expect from surgery and recovery. During the operation, patients should receive short-acting anesthetic agents, no drains, and maintain normothermia. Following the surgery, nasogastric tubes should be avoided, antiemetics used as needed, and oral nutrition, catheter removal, and mobility should be stressed [63]. The broad goal of early mobilization encompasses two important aspects: general mobilization and targeted mobilization. General mobilization encourages the patient to get out of bed as early as possible; to sit, stand, and walk as able; and to allow for limb movements that might prevent venous thromboembolism. Targeted mobilization means the specific organ or body area affected by the surgery should be rehabilitated, ideally using active and passive exercises with the goal of building back strength and improving range of motion. Targeted mobilization should be commenced early and must be continued after the patient is discharged from the hospital.

Clinical pathways

Clinical pathways are process tools designed to help improve postoperative recovery and promote high-quality care. They have been created for specific surgical procedures and can be further tailored to the individual patient. When implemented, clinical pathways have been associated with significantly shorter hospital lengths of stay and fewer in-hospital complications [65]. While both clinical pathways and ERAS protocols are evidence-based guidance designed to improve recovery, ERAS protocols are published and peer-reviewed and set specific outcome criteria, while clinical pathways are not published and may not set outcome criteria. They tend to be a guide to support decision-making efforts in patient care [66]. Regional clinical pathways have been recommended based on the understanding that there are regional, local, and even hospital-based variations in terms of care [67].

Prior to surgery, clinicians should provide preoperative patients with instructions about recovery, particularly in terms of ambulation, movement, and exercise. Take-home materials or videos can be very helpful, such as the “Prep Well” program from the United Kingdom with instructions on self-guided prehabilitation [68]. These prehabilitation programs offer the greatest benefit to the most unfit. For instance, presurgical exercises intended to reduce frailty in surgical patients have been shown to improve their surgical outcomes [69]. In particular, the identification of highly vulnerable patients prior to surgery and building up their physiologic reserve has led to improved outcomes in older, frail individuals [70].

The benefits of prehabilitation and early mobilization can vary by type of surgery. Colorectal surgeries have been extensively evaluated and prehabilitation may be more effective than postoperative rehabilitation in restoring the patient’s baseline level of function [71]. Those with diminished or limited physical capacity before surgery can derive the most benefit from preoperative programs of prehabilitation. The use of chest tubes, urinary catheters, and other lines can be barriers to mobility and must be managed prudently. Pain control also plays an important role in postoperative mobility, but it can be challenging to manage pain using opioid analgesics because of opioid-associated adverse events including opioid-induced constipation [72]. Thoracic surgery patients benefit from early mobilization within 24 hours after surgery [73]. For pancreatoduodenectomy patients, ERAS was not shown to improve outcomes [74]. Several studies on the role of physical activity in bariatric surgery patients produced mixed results [75]. On the other hand, patients undergoing surgery for total hip or knee replacement should be mobilized as early as possible, as a meta-analysis showed a significant 1.8 days reduction in length of stay when patients get up to walk within 24 hours of surgery [66]. However, it is not clear if early ambulation can be associated with a reduction in other complications such as joint dislocation or bleeding risks [66]. A meta-analysis of studies found insufficient evidence to support the best approach to mobility following hip fracture surgery; this review examined weight-bearing exercises, electrical stimulation, treadmill exercises, and resistance training programs [76]. ERAS protocols can be effective in breast surgery; exercise following breast surgery can reduce limb fatigue, seroma, joint arthralgia, lymphedema, and chronic pain, and may improve range of motion [77]. Protocols for at-home exercise should be made available to breast cancer patients [78].

Telehealth exercise programs using smartphone apps or other online technologies have allowed patients at home to benefit from supervised and monitored exercise programs [79]. Clinicians may wish to recommend such programs to selected patients and monitor other programs used by patients for their suitability in the postoperative period.

While early mobilization and postoperative exercise programs may be encouraged, there are numerous barriers that can prevent their use or minimize their effectiveness [80]. Pain can limit early mobilization, particularly any pain that is exacerbated by exercise. Postsurgical drains can impede movement and may prevent exercise. Some patients have surgical complications, orthostatic intolerance, ileus, or delirium that prevents exercise. In addition, postoperative pain control regimens may have side effects that limit exercise, such as nausea, vomiting, and somnolence. Other barriers may include family discouragement and dislike of exercise [80]. Some patients may embark on an exercise plan only to discontinue it because of nausea, vomiting, dizziness, and even syncope. This may be due to orthostatic intolerance, a prevalent stress response to surgery [81]. Postoperative orthostatic intolerance may be managed using preoperative high doses of glucocorticoids (such as >15 mg dexamethasone equivalents). This may enhance analgesia, decrease nausea and vomiting, and relieve early postoperative fatigue, all of which may aid in the initiation of a postoperative mobilization plan [82]. In a prospective observational study of 495 patients undergoing elective cardiothoracic or abdominal surgery, 39% developed orthostatic hypotension after surgery and independent predictors of postoperative orthostatic hypotension were male sex and epidural analgesia [83].

Postoperative pain can itself be an impediment to postoperative rehabilitation; therefore, it is important to provide adequate pain treatment along with educational support to encourage rehabilitation. Patients should be informed about “good pain” and “bad pain” in the course of their rehabilitation and be encouraged to work through tolerable amounts of appropriate pain but to stop the program and alert the clinical team when pain signals something is wrong. Pain on movement needs to be adequately controlled to allow for appropriate exercise, but the pain should not be dulled to the point that the patient is unable to detect “bad pain” from overuse or improper movements. It is important to counsel patients that complete elimination of all postoperative pain may not be helpful (if it is even possible) because rehabilitation and early mobilization are crucial to recovery and they may require awareness of some level of pain. Also, patients should be made aware that "pain trajectory" is a natural process, and with therapy, we may help to speed up the process [84].

## Conclusions

Pain following surgery is normal, expected, and typically follows a predictable trajectory, but a subset of patients will develop CPOP. CPOP may be addressed by adequate perioperative analgesia, control of acute postoperative pain, and early ambulation, mobilization, and rehabilitation. The new paradigm for surgical patients regards patients and their families as active participants in care. Patient education, shared decision-making models, and early rehabilitation can help reduce hospital stays and speed recovery. It is important that acute postoperative pain be interrupted so it does not transition to CPOP, a condition that is more challenging to manage and may be associated with dysfunction and disability.
